# Dietary Fiber, Atherosclerosis, and Cardiovascular Disease

**DOI:** 10.3390/nu11051155

**Published:** 2019-05-23

**Authors:** Ghada A. Soliman

**Affiliations:** Department of Environmental, Occupational and Geospatial Health Sciences, City University of New York, Graduate School of Public Health and Health Policy, 55 West 125th St, New York, NY 10027, USA; ghada.soliman@sph.cuny.edu; Tel.: +1-646-364-9515

**Keywords:** dietary fiber, soluble fiber, insoluble fiber, functional fiber, food groups, cardiovascular disease, the chemical composition of fiber

## Abstract

Observational studies have shown that dietary fiber intake is associated with decreased risk of cardiovascular disease. Dietary fiber is a non-digestible form of carbohydrates, due to the lack of the digestive enzyme in humans required to digest fiber. Dietary fibers and lignin are intrinsic to plants and are classified according to their water solubility properties as either soluble or insoluble fibers. Water-soluble fibers include pectin, gums, mucilage, fructans, and some resistant starches. They are present in some fruits, vegetables, oats, and barley. Soluble fibers have been shown to lower blood cholesterol by several mechanisms. On the other hand, water-insoluble fibers mainly include lignin, cellulose, and hemicellulose; whole-grain foods, bran, nuts, and seeds are rich in these fibers. Water-insoluble fibers have rapid gastric emptying, and as such may decrease the intestinal transit time and increase fecal bulk, thus promoting digestive regularity. In addition to dietary fiber, isolated and extracted fibers are known as functional fiber and have been shown to induce beneficial health effects when added to food during processing. The recommended daily allowances (RDAs) for total fiber intake for men and women aged 19–50 are 38 gram/day and 25 gram/day, respectively. It is worth noting that the RDA recommendations are for healthy people and do not apply to individuals with some chronic diseases. Studies have shown that most Americans do not consume the recommended intake of fiber. This review will summarize the current knowledge regarding dietary fiber, sources of food containing fiber, atherosclerosis, and heart disease risk reduction.

## 1. Introduction

Heart disease is the leading cause of morbidity and mortality in the United States. Cerebrovascular disease (stroke) was the 5th leading cause of death in 2016, according to the National Vital Statistics [1]. To date, statins have been the most effective treatment for lowering blood Low-Density Lipoprotein cholesterol (LDL-C), the major risk factor for atherosclerotic cardiovascular disease [2]. Statins inhibit the 3-hydroxy-3-methylglutaryl-coenzyme A (HMG CoA) Reductase enzyme, which is the last step in the endogenous cholesterol biosynthesis, and thereby decrease blood cholesterol levels. In clinical trials, non-statin drugs had a little utility in the reduction of blood cholesterol due either to tolerability factors or a lack of health outcome [3]. However, statin treatment is costly and is associated with side effects, particularly when high doses are needed. Furthermore, statin non-adherence and discontinuation rates remain high, and many at-risk patients do not achieve optimal LDL-Cholesterol lowering effect with statin as monotherapy [4]. Recently, dietary fiber has been recommended as a dietary change that can be incorporated in addition to statin therapy to increase its efficacy, improve health outcome, and to lower the prescribed dose for statins. A recent meta-analysis of three randomized controlled trials revealed that the addition of dietary gel-forming viscous soluble fiber doubled the efficacy of statins [5]. Additionally, NHANES cross-sectional Data (2003–2006) showed that intake of whole grain, which is rich in dietary fiber, was associated with enhanced effects of statins in lowering blood cholesterol [6]. Historically, crude fibers have been extracted from animal feeds in Germany since 1850 [7]. The benefits of dietary fiber in lowering lipid and cholesterol levels were reported in South African Bantu in 1954 [8]. Observations of the impact of dietary fiber on lipid metabolism were reported in cockerel in 1964 [9]. Fisher et al. (1964) fed cockerel a 5% pectin-supplemented diet for a year and a half and found that these avian sequestered and excreted two times more cholesterol and three times more lipid than cockerel fed the standard diet [9]. The purpose of this review is to highlight the importance of dietary fiber in lowering blood cholesterol and to shed light to the value of soluble gel-forming fiber as an adjuvant to statins in lowering blood cholesterol, which is a hallmark of cardiovascular disease.

## 2. Chemistry and Metabolism of Fiber

Dietary fibers are a complex group of carbohydrates and lignin that are not hydrolyzed by human enzymes and, therefore, are not digested or absorbed in the human body [10]. Dietary fiber is intact in plants and is composed of a complex polymer of phenylpropanoid subunits. Soluble fiber is the edible part of the plant that is resistant to digestion but could be partially or totally fermented by colonic bacteria to short-chain fatty acids in the large intestine. Meanwhile, insoluble fiber passes through the digestive tract intact [11].

Insoluble fiber includes cellulose, some hemicellulose, and lignin. Cellulose is a long linear polymer made of β (1–4)-linked glucose units, and the hydrogen bond between glucose residues gives the 3-dimensional structure of cellulose. Hemi-cellulose is formed of both hexose and pentose sugars with the backbone linked by β (1–4) bonds, while the side chain includes galactose, arabinose, and glucuronic acid linked via β (1–2) and β (1–3) bonds. On the other hand, lignin is formed of phenol polymers that are highly branched with strong intramolecular bonds [11].

Soluble fiber encompasses pectin, gums, mucilage extracted from psyllium husk, β-glucan, and fructans, as well as some hemicellulose. Pectin is a heterogeneous polysaccharide and is composed of unbranched chains of α (1–4)-linked D-galacturonic acid backbone, with chains of pentose and hexoses attached to the backbone. Gums are secreted at the site of plant injury and contain galactose backbone linked by β (1–3) and β (1–6) bonds with side chains of arabinose, glucuronic acid, methyl-glucuronic acid or galactose. Mucilage, found in the plant psyllium, is structurally similar to gums, and is viscous, gel-forming water-soluble fiber containing up to 80% soluble polysaccharide. On the other hand, β-glucans are formed of homopolymers of glucose subunits, while fructans are polymers of fructose including oligofructoses and inulin.

Soluble fiber is resistant to hydrolysis by small intestinal enzymes in humans but is fermented by bacteria to short-chain fatty acids (SCFA) in the large intestine. The production of SCFA leads to alterations in the intestinal microbiota, which contributes to the hypocholesterolemic effects of soluble fiber [12]. Dietary fiber adds bulk to the diet, adsorbs and sequesters cholesterol, and thus decreases hepatic absorption and increases excretion through bile and fecal lipids and bile acids.

## 3. Dietary Fiber and Functional Fiber

Dietary fibers including soluble and insoluble fibers refer to ingredients in plants including non-digestible carbohydrates and lignin. The plant cell wall consists of a primary and secondary wall, which represents most of the content of dietary fiber. Dietary fibers are classified based on their solubility in hot water, water holding capacity (hydration), and viscosity [7,13]. As mentioned above, soluble fibers include viscous fibers such as β-glucans, fructans (inulin, fructooligosaccharides), gum, pectin, mucilage, and non-viscous fibers such as hemicellulose. Soluble fibers absorb water, leading to gel formation, which increases food transit time, delays gastric emptying, decreases nutrient absorption, and slows digestion. Food sources of soluble fiber include vegetables such as carrots, broccoli, onion, and artichokes, and fruits including bananas, berries, apples, and pears, as well as legumes, oats, and barley (Table 1). The insoluble fibers include some hemicellulose, cellulose, and lignin. Unlike soluble fiber, the insoluble fiber decreases transit time and increases fecal bulk, and thus helps to relieve constipation. The insoluble fibers are found in whole grain, wheat, bran, nuts, and seeds, as well as in some fruits and vegetables. While both soluble and insoluble fibers are undigestible, and can be fermented by bacteria using its own enzymes to hydrolyze the fiber, soluble fibers are much more easily fermentable by the gut bacteria, and thus have some prebiotic functions and provide a source of short-chain fatty acids. As such, short-chain fatty acids are rapidly absorbed from the large intestine and can be oxidized for energy production. Absorption of short-chain fatty acids such as propionic acid has been shown to decrease cholesterol synthesis in the liver, leading to decreased blood cholesterol and increased sodium and water absorption into the colonic mucosal cells [14,15]. Short-chain fatty acids also increase the acidification of colon luminal environment, in turn, the acidic pH decreases the solubility of the free bile acids, and increases excretion of bile and at the same time decreases the conversion of free bile acids to secondary bile acids which are more toxic.

On the other hand, functional fibers refer to nondigestible carbohydrates that are either extracted and isolated or synthesized and manufactured, and they have been shown to confer beneficial health effects in humans. Functional fibers include β-glucans, cellulose, chitins and chitosan, fructans, gums, lignin, pectin, polydextrose and polyols, psylliums, resistant dextrins, and resistant starches [7]. Prebiotics are a class of functional fiber that selectively stimulate the activity or growth of beneficial health-promoting bacteria in the colon, mainly lactobacilli and bifidobacteria, and thereby improve the host’s health [18]. To be classified as prebiotics, the fiber ingredients should be resistant to hydrolysis by human enzymes; therefore, they should not be digested or absorbed, they should be resistant to gastric acidity, and able to be fermented by the gut microbiota, and also they should selectively stimulate the activity or growth of healthy intestinal bacteria [18]. Examples of prebiotics include galacto-oligosaccharides, fructooligosaccharides (fructans), and lactulose. The total fiber consumption is the sum of intakes of dietary fiber and functional fiber.

## 4. Recommended Dietary Fiber Intakes

The recommended Dietary Reference Intake (DRI) daily allowance in men aged 19–50 years is 38 g/day and women 25 g/days, and for men ages > 51 is 31 g/day and women ages > 51 is 21 g/day. The recommendation for children ages 1–3 is 19 g/day and ages 4–8 is 25 g/day. For boys, ages 9–13, the DRI recommendations are 31 g/day, and 38 g/days for ages 14–18. For girls ages 9–18, the DRI recommendations are 26 g/day. Although dietary fibers have been shown have several beneficial health effects, the average daily intake for most Americans is 15 g/day, which is much lower than the recommended amount [19]. There is no upper tolerable level for fiber intake, but the tolerance varies by individual, and the most common side effects from overconsumption are bloating and abdominal discomfort.

## 5. Fiber, Blood Cholesterol, and Atherosclerosis

### 5.1. Animal Studies

Studies in rats showed that administration of isomaltodextran was associated with decreased fat absorption compared to the control vehicle, and this effect was observed for up to 6 hours [20]. The authors attributed the mechanism to increased micelle stabilization and enlarged particle size. Our group showed in guinea pigs that animals fed pectin, guar gum, and psyllium exhibited increased LDL-ApoB 100 turnover, which led to the upregulation of hepatic LDL receptors, leading to faster catabolism and clearance [21,22,23,24,25]. Additionally, the hypocholesterolemic effect of fiber was due to the decreased number of secreted VLDL particles, and decreased cholesteryl ester transfer protein (CETP) activity leading to reduced cholesteryl ester in VLDL particles that are transferred to LDL, and at the same time enhanced VLDL and LDL apo B 100 turnover [21]. Studies conducted in several animal models showed the beneficial effects of dietary fibers in the reduction of heart disease risk or reduction in cardiovascular disease mortality. For example, Lo et al. (1987) showed that dietary fiber isolated from soybean was effective in preventing atherosclerosis in rabbits [26].

Similarly, Beakey et al. (1988) reported that grapefruit pectin reduced atherosclerosis in miniature swine [27]. McCall et al (1992) compared the intake of low-cholesterol cellulose (LCC), high-cholesterol psyllium (HCP), and high-cholesterol cellulose (HCC) in African green monkeys for 3.5 years and reported that both LCC and HCP significantly reduced blood cholesterol than HCC and that dietary psyllium decreased total blood cholesterol by decreasing LDL cholesterol synthesis [28,29]. Roach and Topping et al. (1990, 1992) reported that the combination of oat bran and fish oil decreased blood cholesterol levels in rats [30,31]. In addition, Wilson and colleagues found that barley and insoluble fibers had a hypocholesterolemic effect in Syrian Gold Hamsters [32,33,34]. Similarly, several investigators have documented the beneficial effects of certain types of dietary fiber in reducing blood cholesterol in mice [35,36,37,38,39,40,41]. Taken together, studies in animal models reveal the importance of both soluble and insoluble fibers in lowering blood cholesterol, attenuating atherosclerosis, and decreasing heart disease risk.

### 5.2. Human Studies

#### 5.2.1. Observational Studies

Several cohort studies have investigated the intake of dietary fiber and coronary heart disease, as well as cardiovascular disease, in the U.S. and globally [42,43,44,45,46,47,48,49,50,51,52,53]. These studies documented a protective effect of dietary fiber on the reduction of heart disease. Pereira et al. (2004) conducted a meta-analysis of ten cohort studies with 6–10 years follow-up [44]. The group reported an inverse relationship between dietary fiber intake and the risk of cardiovascular disease with a Relative Risk (RR) of 0.84 (95% CI, 0.70–0.99). However, an additional 10 gram per day increment of fiber intake was not statistically significant, with a relative risk of 1.0 (95% CI 0.88–1.13). Similarly, Threapleton and colleagues (2013) performed a meta-analysis study to determine the dose–response relationship between dietary fiber intake and the risk of cardiovascular disease [54]. The investigators reported that the pooled protective effect for each 7 g/day increase in fiber intake was RR = 0.91 (CI 0.87 to 0.94). However, higher doses of fiber had a larger confidence interval around the mean, and the results were less reliable [54]. Additionally, Buil-Cosiales and colleagues (2014) documented that the intake of fiber from fruit was associated with decreased all-cause mortality in the Prevencion con Dieta Mediterranea (PREDIMED) study (Hazard Ratio 0.59, 95% CI = 0.44, 0.78) [55]. Over the last three decades, several investigators have reported the benefits of dietary fiber from a variety of food sources in decreasing the risk of cardiovascular diseases [54,56,57,58,59,60,61,62,63,64]. Therefore, based on the evidence, it appears that fiber consumption with moderation is recommended.

The main limitations of observational studies include bias [65] and confounding variables [66], as well as showing associations and correlations rather than causation. Selection bias can be encountered in cohort studies due to informative censoring and measurement errors, and can be found in case-control studies due to inappropriate selection of controls. At the same time, confounding may occur due to the co-existence of exposures leading to the same health outcome. Confounding is difficult to account for unless all common causes of exposure and their link to the disease outcomes are known. Therefore, randomized control trials studies are conducted to address the causality of fiber in reducing cardiovascular disease and mortality and to eliminate confounders, measurement errors, and selection bias.

#### 5.2.2. Randomized Control Trials

As mentioned above, observational studies have suggested that the intake of dietary fiber was associated with decreased risk of heart disease. Randomized control trials are employed to determine the causality of dietary fiber on improving the lipid profile. As such, several randomized control trials investigated the effects of different fibers on atherosclerosis and heart disease. Li et al. compared the intake of quinoa-enriched bread (20 g quinoa flour) to the intake of refined wheat in 37 overweight healthy men aged 35–70 years with BMI > 25kg/m^2^ in a four-week crossover design with four weeks washout period. The authors reported that after four weeks, blood cholesterol and blood glucose were lower than the baseline in both groups, but there was no difference between the participant’s groups which consumed quinoa versus the group that consumed 100% refined wheat. The authors attributed the lack of significance to the short follow-up period [67]. Another randomized controlled crossover trial for five weeks in 30 participants with mild hypercholesterolemia compared the intake of β-glucan with a control diet and reported the reduction of total cholesterol with β-glucan groups, but no effect on cholesterol synthesis or absorption. The authors speculated that lowering blood cholesterol levels were attributed to increased bile acid synthesis [68]. Similarly, a randomized, controlled, open-label, parallel group study in Asian Indians compared the intake of 3 grams of soluble fiber from oats with a control group maintaining a routine diet for four weeks in healthy adults male and female participants (blood cholesterol 200 mg/dL-240 mg/dL) [69]. The investigators reported a significant reduction in total blood cholesterol in the intervention group versus the control group, which consumed athe usual diet (8.1% versus 3.1%, *p* < 0.02), as well as LDL-cholesterol (11% vs. 4.1%, *p* < 0.04). Taken together, the results from these short-term studies showed a trend of decreased total and LDL cholesterol and improved cardiovascular biomarkers.

The variability in outcome was attributed to the short-term nature of the studies. Therefore, longer-term intervention was suggested to confirm these findings. Yen et al. investigated the long-term (8 weeks) effects of a diet-controlled study on supplementation with functional fibers, isomalto-oligosaccharides, in 13 subjects with constipation. The protocol consisted of four weeks placebo and two periods of four-week supplementation (total of 8 weeks) with isomalto-oligosaccharides, and a four-week post period. The investigators reported improved colonic microflora profile during treatment only and reduced LDL-cholesterol and total cholesterol during the intervention, as well as during the follow-up period [70]. A 6-month randomized controlled intervention study with a one-year follow-up investigated the impact of rice bran extract consumption in post-menopausal Vietnamese women (*n* = 30/group). The participants in the intervention group received 50 mg of acylated steryl glucosides (PSG) brown rice bran extract (6 capsules), while the placebo group received six capsules of corn oil. The results showed a significant reduction in LDL cholesterol in the intervention group compared to the placebo group (from 163 ± 25.3 mg/dL vs. 135.9 ± 26.8mg/dL), and decreased TNF α, inflammatory marker (from 6.6 ± 5.5% to 4.72 ± 6 respectively [71]. Another long-term one-year pre-post intervention trial in 66 participants with hyperlipidemia found that following a plant-based diet for one year led to a significant reduction in blood pressure and LDL cholesterol that was maintained at the one-year follow-up [72]. The plant-based diet consisted of a diet high in soy protein (22.5 g/1000 kcal), viscous fibers (10 gm/1000gram), and almond (23 gm/1000g). Similar findings were reported in other studies in which participants consumed a plant-based diet with the addition of monounsaturated fatty acids or two levels of dietary advice to promote hypercholesterolemia control [72,73,74,75,76]. Taken together, based on the long-term randomized control trials, there is strong evidence to indicate that intake of dietary soluble fiber is associated with improved lipid profile, inflammatory markers, and improved health.

## 6. Other Fiber Functions

Dietary fiber has several protective effects against chronic diseases, including cardiovascular disease, diabetes, metabolic syndrome, inflammatory bowel syndrome, diverticular disease, obesity, and colorectal cancer in the age-adjusted analysis [77,78,79,80,81,82,83,84]. For example, insoluble fiber binds to and adsorbscarcinogens, mutagens, and toxins, and therefore, prevents their harmful effects to the body, by preventing the toxins absorption and targeting them for elimination [83,85,86]. Other fiber properties include delayed colonic transit time, prolonged post-meal satiety and satiation, and induction of cholecystokinin satiety hormone [87,88]. The Academy of Nutrition and Dietetics position on fiber intake is to increase consumption of whole grains, fruits and vegetables, nuts and legumes and that dietary fiber is associated with risk reduction of type 2 diabetes, cardiovascular disease, and select cancer types [89].

## 7. Summary

Dietary fiber can be used as a dietary change to complement statin monotherapy in lowering total and LDL-Cholesterol and to reduce the prescribed dose of statin, decrease the side effects, and improve drug tolerability. Soluble and insoluble dietary fibers in whole foods have multiple non-nutritive health effects thathelp improve the lipoprotein profiles, and have no caloric value, and thus could be part of a healthy eating pattern. The abundance of dietary fiber in whole grain protein food, fruits, and vegetables, makes them attractive targets for disease prevention and reduction of risk of atherosclerosis and cardiovascular disease.

## Figures and Tables

**Table 1:** Select sources of dietary fiber in the food groups.

Fiber Content in The Food Groups **	**Food Item**	* NDB ID	**Grams/Cup**
**1. Grain**	Corn bran, crude	20015	60
	Barley, hulled	20004	31.8
	Rye flour, dark	20063	30
	Wheat bran, crude	20077	24
	Rice bran, crude	20060	24.8
	Bulgur, dry	20021	17.5
	Oats	20038	16
	Sorghum grain	20067	12.9
	Cereal, ready to eat (granola)	08037	10.9
	Cornmeal, self-rising	20324	10.7
	Wild rice, raw	20088	9.9
	Pasta, whole grain	20135	9.2
	Couscous, dry	20028	8.7
	Rice, brown, long grain	20036	6.7
**2. Protein Foods**	Beans, kidney, all types	16027	45.8
	Soybeans, mature, roasted	16410	30.4
	Peas, green, split, raw	16085	43
	Seeds, sesame seed	12029	21.6
	Lentils, pink or red, raw	16144	20
	Nuts, almond, oil roasted	12065	16.5
	Peanuts, oil roasted	16389	13.5
	Chickpeas (garbanzo beans) canned	16360	10.6
**3. Fruits**	Passion fruit, purple, raw	09231	24.5
	Blueberries, canned, heavy syrup, drained	09353	15
	Figs, dried, uncooked	09094	14.6
**The Fiber Content in the Food Groups**	**Food Item**	* NDB ID	**Grams/Cup**
**3. Fruits**	Peaches, dried, sulfured, uncooked	09246	13
**(Continued)**	Plums, dried (prunes), uncooked	09279	12.4
	Raisins, seeded	09299	11.2
	Apricots, dried, sulfured, stewed with added sugar	09034	11.1
	Avocado, raw	09037	10.1
	Prunes (dried plum)	09293	9.4
	Guava, common, raw	09139	8.9
	Oranges, raw with peel	09205	7.7
	Plantain, green, raw	09542	5.9
	Kiwi fruit, green, raw	09148	5.4
	Blueberry, raw	09050	3.6
	Apple, granny smith, raw	09502	3.1
	Strawberry, raw	09316	3
	Peaches, yellow, raw	09236	2.3
	Plum, raw	09279	2.3
**4. Vegetables**	Potatoes, mashed, dehydrated granules	11380	14.2
	Mixed vegetables	11579	9.3
	Sweet potatoes, cooked, boiled	11510	8.2
	Edamame, frozen, prepared	11212	8.1
	Artichokes, frozen, cooked, boiled, drained	11703	7.7
	Collard, cooked, boiled	11162	7.6
	Tomato (sun-dried)	11955	6.6
	Brussel Sprouts, frozen, chopped	11093	6.4
	Corn (yellow, dried)	35183	5.8
	Broccoli, frozen, chopped	11093	5.5
	Squash, winter, hubbard, raw	11489	4.5
	Carrots, cooked (frozen)	11131	4.8
	Pea, raw	09252	4.3
	Gums, guar, seed gums	42281	21

* Nutrient Database (NDB) source of data is the US Department of Agriculture, USDA Food Composition Databases; Software developed by the National Agricultural Library v.3.9.5.1_2019-01-29. ** Classification of the Food Groups: Grains, Food Group, Fruits, Vegetables, and Dairy) [16,17].
